# Evaluation of optimal Zernike radial degree for representing corneal surfaces

**DOI:** 10.1371/journal.pone.0269119

**Published:** 2022-05-26

**Authors:** Pooria Omidi, Alan Cayless, Achim Langenbucher

**Affiliations:** 1 Department of Experimental Ophthalmology, Saarland University, Homburg/Saar, Germany; 2 School of Physical Sciences, The Open University, Milton Keynes, United Kingdom; University of Toronto, CANADA

## Abstract

Tomography data of the cornea usually contain useful information for ophthalmologists. Zernike polynomials are often used to characterize and interpret these data. One of the major challenges facing researchers is finding the appropriate number of Zernike polynomials to model measured data from corneas. It is undeniable that a higher number of coefficients reduces the fit error. However, utilizing too many coefficients consumes computational power and time and bears the risk of overfitting as a result of including unnecessary components. The main objective of the current study is to analyse the accuracy of corneal surface data modelled with Zernike polynomials of various degrees in order to estimate a reasonable number of coefficients. The process of fitting the Zernike polynomials to height data for corneal anterior and posterior surfaces is presented and results are shown for normal and pathological corneas. These results indicate that polynomials of a higher degree are required for fitting corneas of patients with corneal ectasia than for normal corneas.

## Introduction

The cornea is one of the most influential optical components of the human eye, being responsible for about two-thirds of the eye’s refractive power. The cornea is characterized by its anterior and posterior surfaces. Although the standard shape of the cornea is a prolate spheroid, there is a wide diversity of shapes in the human cornea. Corneal deformations are generally caused by corneal diseases such as keratoconus. Therefore, accurate evaluation and simulation of corneal surfaces are mandatory.

The shape measurement of the anterior and posterior surfaces of the cornea can be performed by non-destructive instruments such as optical coherence tomography devices. For the purpose of modelling the corneal surfaces, one can utilize Zernike polynomial expansion (named after Fritz Zernike, who proposed them in 1934 [1]). The first fundamental obstacle that arises in applying Zernike polynomials is to determine the appropriate number of terms. Vision researchers typically use the first 15 Zernike terms [2]. However, the question is how many Zernike terms are appropriate to describe the refractive properties of any cornea that has more aberration in its surfaces without any loss in profitable data [2, 3].

In this paper, we first describe our data acquisition system and mathematical method. Then we demonstrate the simulation outcomes on two different corneas, and present a statistical approach to 30 different eyes with diverse circumstances. Finally, we discuss our technique to determine the appropriate number of Zernike terms to describe corneal surface shape properly.

## Materials and methods

### Measurement setup

In order to measure corneal shape, we used a 3D swept-source OCT setup CASIA2 (TOMEY Inc., Nagoya, Japan). CASIA2 has an axial resolution of 10 μm with a scanning range of 16 mm in diameter and maximum penetration depth of 13 mm.

### Data collection

For the present study, we used clinical records of patients who were admitted to the Saarland University Clinical Centre (Homburg, Germany). The study was registered at the local ethics committee of the Medical Association of Saarland (Ärztekammer des Saarlandes, 157/21).

A total of 15 normal volunteers and 15 patients were enrolled in the study. The volunteers show a normal corneal tomography, and the patients show corneal ectasia (keratoconus). These patients were in different stages of the Belin Ambrosio keratoconus severity classification (from 0 to 4). This study was carried out on native corneas without any history of eye surgery such as corneal cross-linking or intracorneal ring (segment) implantation.

### Data selection

CASIA2 uses polar coordinates to provide several types of tomographic data such as elevation, refractive, keratometric, etc., from the anterior and posterior surfaces of the cornea.

The CASIA2 software provides raw data files in both CSV and DAT formats. Each data set has 16 meridians with 16 mm of diameter, with each meridian containing 800 data points. This study was restricted to the central 8 mm zone because this represents the average adult cornea diameter under the typical clinical conditions. It should be noted that CASIA2 considers a tangent plane to the centre of the anterior surface as a reference for measuring the height of each point.

All of the data selection procedure and data analysis was carried out using MATLAB R2019b software.

### Zernike polynomials

Zernike polynomials are a set of orthogonal polynomials defined on a unit circle. These polynomials are a product of angular functions and radial polynomials [3]. Since CASIA2 obtains data in polar coordinates, it is advantageous to use polar Zernike functions instead of Cartesian functions because conversion from polar to Cartesian coordinates may affect the data as a result of internal corrections. The radial polynomials are formulated from the Jacobi polynomials and the angular functions are basic functions for the two-dimensional rotation group [4]. Commonly, vision researchers utilize two dimensional Zernike expansions in terms of radial and azimuthal parameters, introduced by Noll [5]. In the present study, Noll notation has not been used, hence the value of the radial polynomials has more priority for us.

The Zernike polynomials are defined as [5]

$$Z_{p}(\rho ,\theta )=\left\{ \begin{array}{c} \sqrt{2(n+1)}R_{n}^{m}(\rho )\cos(m\theta ),evenp,m\neq 0\\ \sqrt{2(n+1)}R_{n}^{m}(\rho )\sin(m\theta ),oddp,m\neq 0\\ \sqrt{2(n+1)}R_{n}^{0}(\rho ),m=0 \end{array}\right.$$

Where *n* is the radial degree, *m* is the azimuthal frequency, and

$$R_{n}^{m}=\sum_{s=0}^{(n-m)/2}\frac{{\left(-1\right)}^{s}(n-s)!}{s!(\frac{n+m}{2}-s)!(\frac{n-m}{2}-s)!}\rho^{n-2s}.$$

The values of *n* and *m* are always integral satisfy *m*≤*n* and *n*−|*m*| = even. The index *ρ* is a mode ordering number and is a function of *n* and *m*.

To reconstruct the surface from extracted Zernike terms, height data from corneal surface in polar coordinates can be modelled by a superposition of weighted Zernike polynomials [5].

$$h(\rho_{d},\theta_{d})=\sum_{p=1}^{P}a_{p}Z_{p}(\rho_{d},\theta_{d})+\varepsilon_{d},d=1,\ldots ,D,$$

where $h(\rho_{d},\theta_{d})$ is corneal surface height, $Z_{p}(\rho_{d},\theta_{d})$ is the *p*-th Zernike polynomial sampled from $Z_{p}(\rho ,\theta )$ at points $\left(\rho_{d},\theta_{d}\right)$. Also *ρ* is normalized distance from the origin, *θ* is angle and *ε* is the deviation of modelled data from raw data.

### Fitting Zernike polynomials

After clipping height data from the periphery (outside the 8 mm central region), we fitted normalized Zernike functions to the topographic shape of the cornea using a least squares regression method to find the best fit for a set of data points. The normalization factor of orthogonal Zernike functions is defined as:

$$N=\sqrt{\frac{\left(2-\delta_{m0})(n+1\right)}{\pi }}.$$

For continuous data points the integral of ${\left({rZ}_{}(\rho_{},\theta_{})\right)}^{2}$ over the unit circle is unity. In this study, the data acquisition resolution is sufficient to consider the discrete data points as continuous and to neglect the mathematical error in the normalization factor.

Our goal is to find a reasonable balance between a low fit error (high number of Zernike polynomials) and avoidance of complexity of the surface (low number of Zernike polynomials).

To validate the method, a surface can be reconstructed with a limited number of calculated Zernike coefficients. The fit error is then calculated by subtracting the reconstructed height data from original height data.

**Fig 1:**
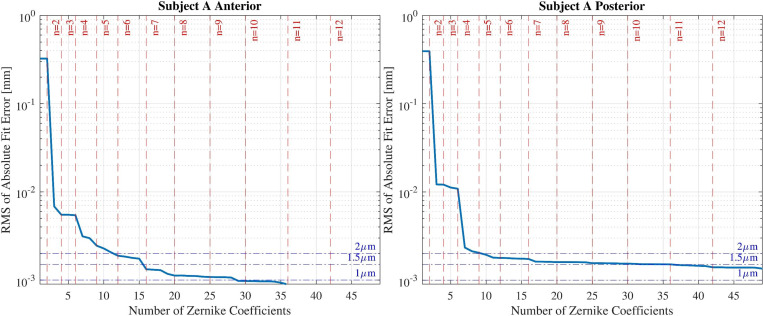
The results of fitting Zernike polynomials to corneal height for subject A. The horizontal dashed lines indicate the radial degree of fitted Zernike terms.

This procedure is repeated each time by adding more coefficients and computing the root mean square of the fit error. For a better examination of the fit error behaviour, The RMS of the absolute value of fit error is plotted as a function of the number of fitted Zernike terms. This diagram (as shown in Fig 1) allows us to determine the reasonable number of Zernike coefficients which represents the refracting surfaces appropriately. In this diagram, vertical and horizontal lines are plotted as threshold indicators for Zernike radial degrees and RMS of the fit error value respectively.

The criterion for choosing the appropriate radial degree is based on the volume of changes in the fit error value by adding more Zernike terms. In other words, an appropriate radial degree is a degree at which adding more Zernike coefficients does not cause a considerable improvement in the fit performance.

The next step is to reconstruct each surface using the estimated radial degree. To calculate the difference between the reconstructed surface and the original one, reconstructed height data are subtracted from the original height data.

To evaluate the performance of the composed height data in different radial zones, we have reconstructed the height data with the selected degree of coefficients at different radii and calculated the mean, median, and maximum from the absolute value of the fit error each time.

## Results

This method was used to estimate the appropriate number of Zernike terms for corneal topography in 2 clinical examples. The first example uses eye measurement data from a normal subject (subject A). For the second example, a subject with mild keratoconus at the corneal surface was selected (subject B). For statistical analysis, the same procedure was then repeated for 15 normal corneas, and also for 15 corneas with various amount of corneal ectasia.

Fig 1 illustrates the RMS of the absolute value of fit error over the number of Zernike terms representing the height data of the anterior and posterior surfaces of subject A. Each radial degree of Zernike terms is denoted by dashed red vertical lines. The blue horizontal lines indicate the RMS value threshold.

**Fig 2:**
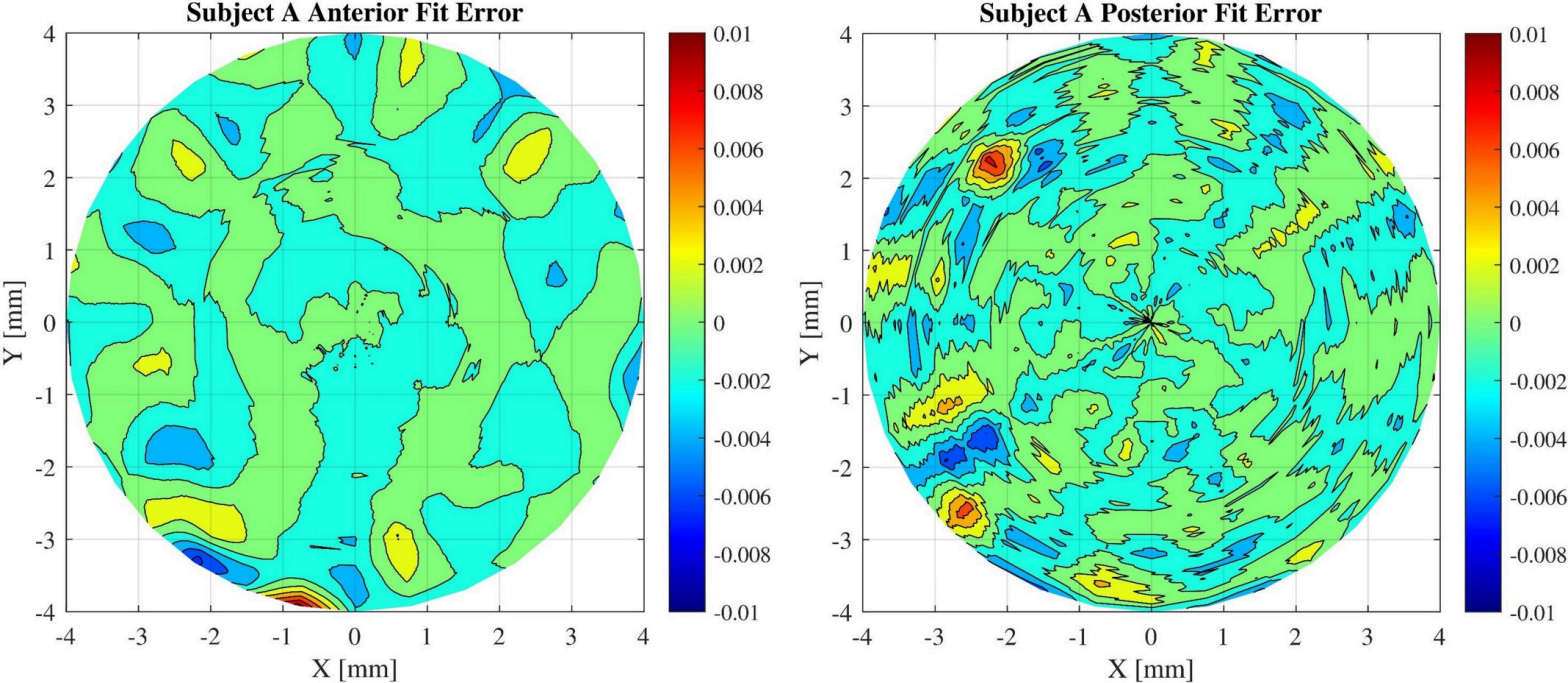
Error values of the fitted corneal surfaces. Both reconstructed anterior and posterior surfaces modelled with Zernike radial degree of 7.

From the graphs a radial degree of 7 is estimated for both anterior and posterior surfaces of subject A cornea. The reason for choosing this radial degree is that there is no significant decrease in RMS value with addition of more Zernike components. Each surface is reconstructed using the estimated radial degree. Fig 2 displays the residuals calculated at each coordinate point and displayed as a contour map.

**Fig 3:**
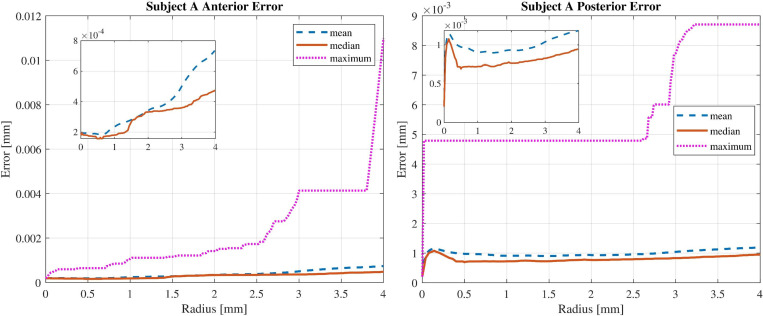
Error analysis for anterior and posterior surfaces modelled with radial degree of 7. Mean, median and maximum values are denoted by dashed, solid, and doted lines.

Fig 3 shows the mean, median and maximum amounts of absolute value of fit error for variations of the central zone diameter for subject A.

**Fig 4:**
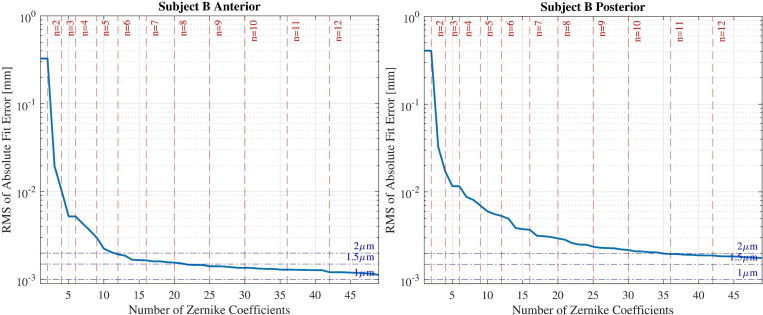
The results of fitting Zernike polynomials to corneal height for subject B. The horizontal dashed lines indicate the radial degree of fitted Zernike terms.

The same procedure was repeated with corneal height data for subject B. Fig 4 illustrates the RMS of the absolute value of fit error over the number of fitted Zernike terms on subject B corneal surfaces.

**Fig 5:**
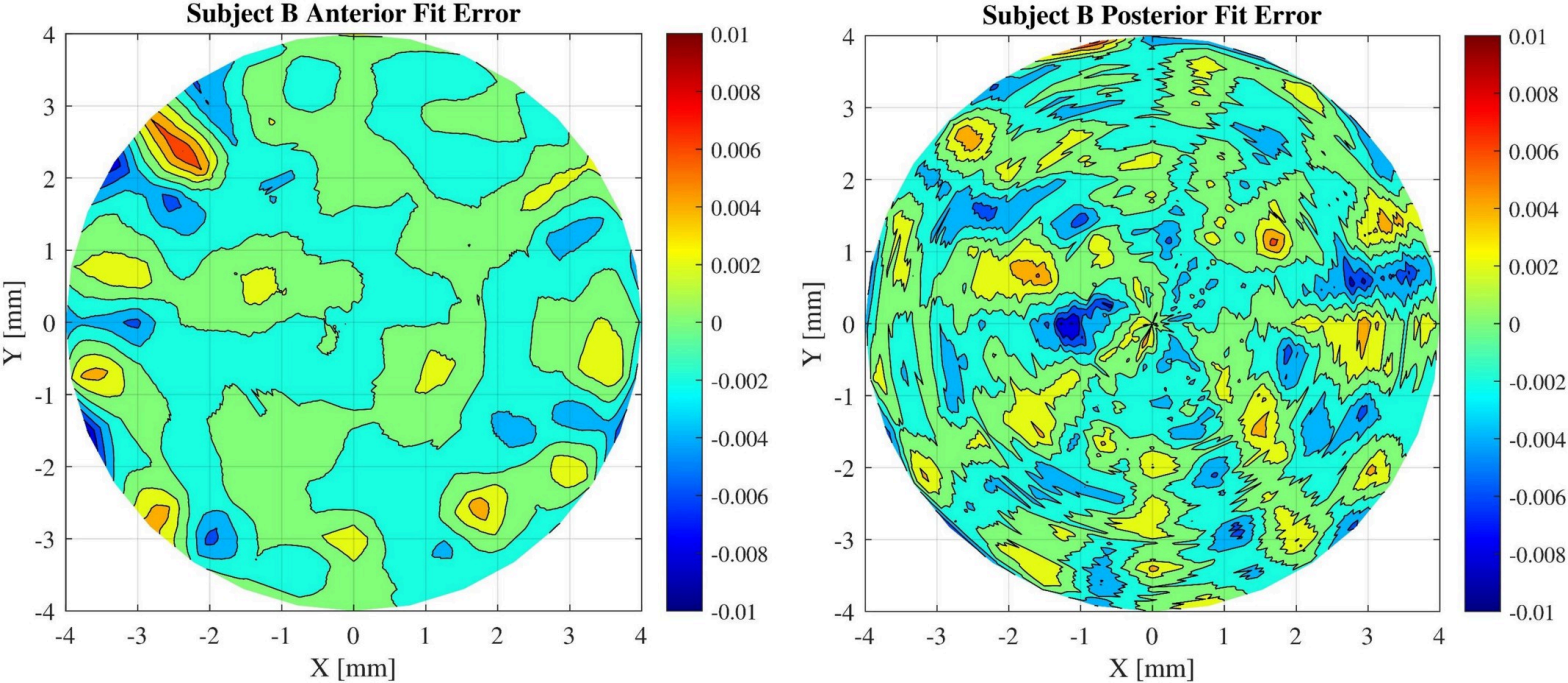
Error values of the fitted corneal surfaces. Reconstructed anterior and posterior surfaces modelled with Zernike radial degree of 7 and 9 respectively.

From the results, radial degrees of 7 and 9 were selected for the anterior and posterior surfaces. Fig 5 displays the residuals at each point of coordinates for subject B.

**Fig 6:**
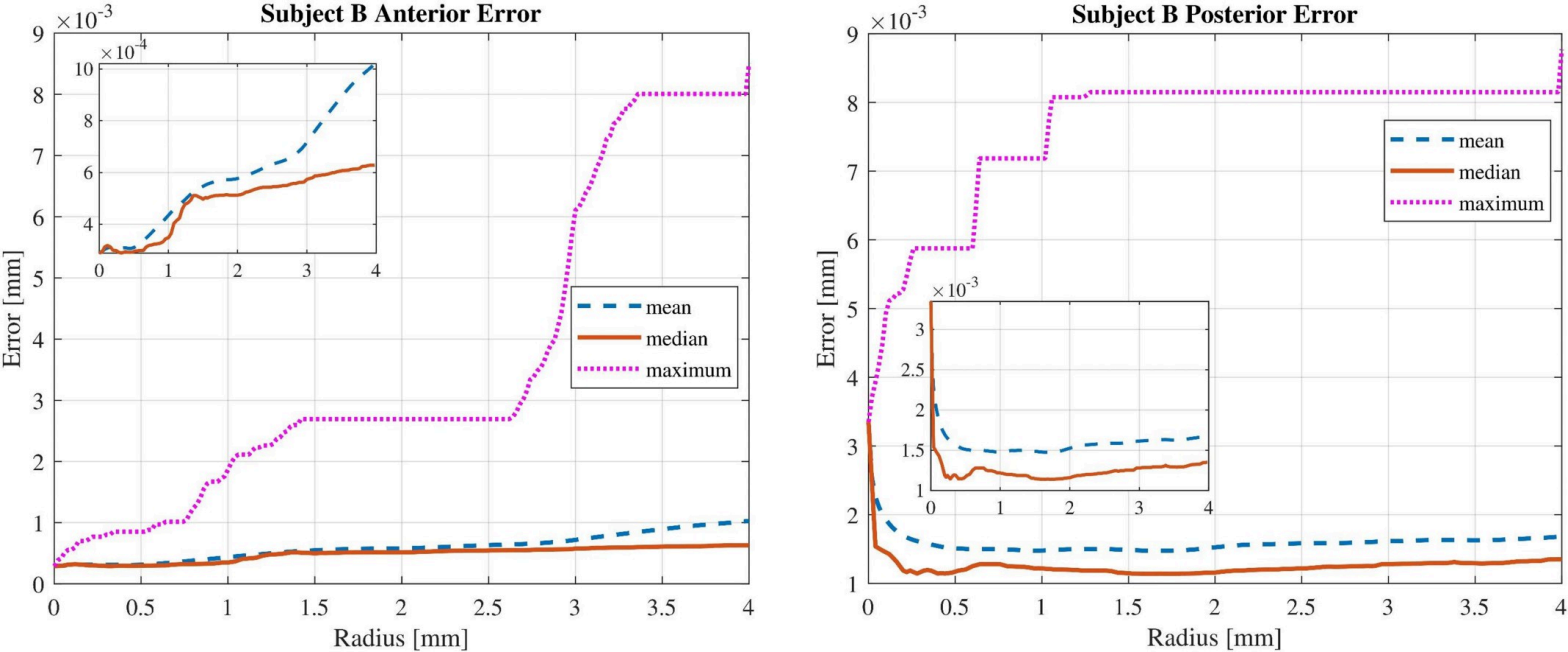
Error analysis for anterior and posterior surfaces modelled with radial degree of 7 and 9 respectively. Mean, median and maximum values are denoted by dashed, solid, and doted lines.

Finally, Fig 6 shows the mean, median and maximum absolute value of the fit error for variations of the central zone diameter for subject B.

The strategy of OCT data processing was used to analyse 30 subjects with a variety of corneal conditions. The results are presented in the next subsection.

### Evaluation of a series of normal and patients

In this subsection, we present the results of our method as tested on 15 normal and 15 patients with various amount of corneal ectasia. Table 1 provides information on the normal subjects and the radial degrees required for modelling the anterior and posterior surfaces of their corneas. Appropriate radial degree for anterior surface denoted by n_A_ and for posterior surface denoted by n_P_.

**Table 1:** Appropriate radial degree of fitted Zernike polynomials for normal subjects.

Subject	Age	Sex	OD/OS	n_A_	n_P_
1	53	Female	OD	7	8
2	30	Female	OS	7	7
3	26	Female	OD	6	8
4	27	Male	OS	6	7
5	27	Male	OD	7	7
6	22	Male	OD	6	7
7	29	Male	OD	7	7
8	28	Female	OD	7	8
9	28	Female	OS	6	7
10	72	Male	OD	5	7
11	45	Female	OS	6	7
12	27	Male	OD	7	7
13	71	Male	OD	7	8
14	35	Female	OD	5	6
15	53	Female	OS	7	7

Similarly, Table 2 provides information on the keratoconus subjects and the radial degrees required for modelling the anterior and posterior surfaces of their corneas.

**Table 2:** Appropriate radial degree of fitted Zernike polynomials for keratoconus subjects.

Subject	Age	Sex	OD/OS	n_A_	n_P_	Classification stage
1	61	Female	OS	7	9	1
2	25	Male	OD	7	9	1
3	42	Male	OS	8	8	2
4	42	Male	OD	7	8	1
5	33	Male	OD	7	9	1
6	28	Male	OD	6	8	1
7	64	Male	OS	8	10	3
8	64	Male	OD	9	11	3
9	14	Female	OD	8	9	2
10	63	Female	OS	7	9	2
11	45	Female	OD	8	8	2
12	80	Female	OD	7	8	1
13	59	Male	OS	9	9	3
14	53	Male	OS	8	8	2
15	18	Male	OS	8	9	2

## Discussion

From Figs 1 and 4 it can be concluded that fitting more Zernike polynomials to the height data of cornea decreases the fit error, up to a certain point. After this point there is no significant change in the fit error value with increasing number of fitted Zernike terms and the slope of the changes gradually tends to zero. Therefore potential overfitting may cause the addition of more measurement noise to the modelled surface. For that reason there should be a balance between accuracy and noise elimination criteria of the modelled surface.

Another result that can be observed from the figures is that the amount of fluctuation of the surface height in the posterior surface is greater than that for the anterior surface of the cornea. This behaviour can be observed in OCT devices, where the amount of white noise increases when deeper structures are measured [6]. Since the optical path difference between the sample and the reference mirror in deeper layers increases, this leads to decrease of contrast. As a result, it can be deduced from Figs 2 and 5 that the posterior surface shows more error fluctuation in its topographic shape and the fit error value of the posterior surface is higher in comparison to the anterior surface.

By analysing the results, it can be concluded that simulated surfaces with finite Zernike polynomials have more similarity in the centre rather than in the periphery. Moreover, the absolute value of fit error increases with increasing zone diameter. It means that the fitting quality has a radial behaviour. This phenomenon can be explained from two perspectives. First, the sparse sampling in the periphery and the denser sampling in the centre. Second, signal to noise ratio decreases if the OCT beam meets the surface more obliquely in the periphery of the cornea.

As expected, the radial degree required for modelling corneas in patients with corneal ectasia is more than for normal corneas. The results in Tables 1 and 2 support this hypothesis.

### Limitations of this study

Our sample did not include eyes with history of corneal surgery and there are only two groups of normal and keratoconic eyes. So it can be concluded that there is a lack of generality in the samples.

This study has the character of a pilot study and our aim was to show the applicability of this concept rather than to study the difference between normal and keratoconic corneas. Therefore, we did not perform a power analysis and the number of samples is limited.

## Supporting information

S1 Data (XLSX)

S2 Data (XLSX)
